# Supplementary material for: Co-Occurrence of Congenital Aniridia Due to Nonsense PAX6 Variant p.(Cys94*) and Chromosome 21 Trisomy in the Same Patient
Source: Int J Mol Sci. 2023 Oct 24;24(21):15527. doi: 10.3390/ijms242115527 (PMC10650867; doi:10.3390/ijms242115527)
Supplement: Supplementary file 1 [file ijms-24-15527-s001.zip › ijms-2661198-supplementary.pdf]

Supplementary Table S1. Dynamic clinical examinations of the patient with congenital aniridia and Down syndrome.

| Age | Ophthalmologic examination | Neurologic examination | Cardiologic examination |
|---|---|---|---|
| 3 month | The patient exhibits floating movements of the eyeballs. In terms of ocular anatomy, there is notable enlargement in the size of the cornea, with a diameter of 10-11 mm in the right eye (OD) and 13 mm in the left eye (OS). In the center of the cornea, there is evident bullish tissue degeneration accompanied by a cloud-like opacity. Moreover, the patient presents with complete aniridia, a condition characterized by the absence of the iris. Due to these conditions, it is not possible to observe the eye fundus. | | The patient has been diagnosed with a restrictive atrial septal defect, as well as a grade 1 deficiency in the mitral valve. Additionally, there is an open ductus arteriosus, though it is not accompanied by significant abnormality of heart hemodynamics. |
| 9 month | The patient displays short-term fixation within a distance of 50 cm. There is also a small-scale horizontal nystagmus present. Furthermore, there is an enlargement in the size of the cornea, with a diameter of 12.5 mm in the right eye (OD) and 15.0 mm in the left eye (OS). These findings are indicative of buphthalmos. Additionally, there is a keratopathy of grade 2-3. | The patient exhibits several distinctive facial features, including a depressed nasal bridge, hypertelorism (widely spaced eyes), antimongoloid slant, and epicanthal folds of the eyelids. Other facial characteristics include an open mouth, macroglossia (enlarged tongue), and hypersalivation.<br>Physical measurements include a head circumference of 40.5 cm and a chest circumference of 41 cm.<br>During examination of cranial nerves, facial hypomimia (reduced facial expressions) is noted, along with asymmetry in eye slits due to the left eyeball enlargement.<br>The patient displays horizontal nystagmus, which involves involuntary eye movements.<br>Muscle tone is generally reduced throughout, but muscle strength is deemed sufficient. There are brisk tendon reflexes, and the arms and feet exhibit lower temperature. The skin has a marble-like appearance.<br>Sensibility and pelvic functions remain preserved. | |

| | | | |
|---|---|---|---|
| | | There is an excess of movement noted in the hip joints.<br>Psychomotor development is delayed, although there are no evident cerebral symptoms or signs of ataxia (lack of muscle coordination). | |
| 12 month | The patient demonstrates the ability to track a toy with both eyes (OU).<br>In both eyes (OU), there is buphthalmos, indicating an enlargement of the eyeballs.<br>In the right eye (OD), tonometry measures between 28-30 mmHg, indicating elevated intraocular pressure.<br>In the left eye (OS), tonometry measures between 23-24 mmHg, indicating relatively lower but still elevated intraocular pressure.<br>Regarding the cornea:<br>In the right eye (OD), the cornea is transparent but displays megalocornea, which is an enlarged cornea.<br>In the left eye (OS), there is partial cloudiness and mild edema present in the cornea. | The patient's reaction to examination is positive.<br>There are no evident general cerebral or meningeal symptoms, and sleep and appetite remain undisturbed.<br>Regarding cranial nerves:<br>There is hypomimia (reduced facial expressions), along with asymmetry in the eye slits.<br>Horizontal nystagmus is observed.<br>An unstable converging strabismus is noted.<br>Muscle tone is generally reduced across the body, and there are brisk tendon reflexes without any signs of pathology.<br>In terms of motor development:<br>The patient is currently crawling but has not yet started walking or sitting independently.<br>She does not use specific gestures to communicate her desires and preferences. Instead, she begins to twist her head to express herself.<br>Regarding speech development:<br>Speech consists of separate, indistinct syllables and monotonous sounds without active and clear babbling. | |
| 18 month | The patient now exhibits spatial vision within a range of 70 cm in both eyes (OU), indicating an improvement.<br>Adnexal development has progressed correctly.<br>In the left eye (OS), there is a milder presentation of buphthalmos compared to previous observations. | The patient is responsive to her parents' calls, engages in games, and reacts to verbal communication. She demonstrates active crawling but has not yet progressed to standing or walking. With support, she is capable of assuming a kneeling position.<br>Muscle tone is moderately reduced, indicating some level of muscle relaxation. | |

| | | | |
|---|---|---|---|
| | Regarding the cornea:<br>In the right eye (OD), the cornea remains transparent but exhibits megalocornea, indicating enlargement.<br>In the left eye (OS), there is partial cloudiness, milder edema, and loose epithelium.<br>In the right eye (OD), the anterior chamber of the eye is slightly deeper than it should be. The lens remains transparent.<br>Upon fundus examination of the right eye (OD):<br>The optic nerve disc appears pale with sharp contours, reduced in size, and excavated by 0.4 due to glaucoma.<br>The course and caliber of the vessels appear normal.<br>The macula zone reflex is blurred, while the visible periphery appears normal.<br>In the left eye (OS), there is a presence of fleur behind it. | In terms of speech development, she is able to pronounce individual syllables. | |
| 2 y.o. | In terms of visual acuity:<br>The right eye (OD) exhibits good spatial vision up to 60 cm.<br>The left eye (OS) has spatial vision limited to 20 cm.<br>Both eyes together (OU) show heterotropia, indicating misalignment of the eyes.<br>Extraocular movements are normal in all directions.<br>Tonometry readings are as follows:<br>Right eye (OD): 21 mmHg<br>Left eye (OS): 24 mmHg<br>Regarding the corneas:<br>In the right eye (OD), there is no negative change, with a diameter of 12.0 mm. | | As of June 26, 2020, an echocardiogram revealed an open arterial duct measuring 3.7 mm in size. Subsequently, on July 3, 2020, endovascular closure of the open arterial duct was successfully performed using an occluder. The patient is currently not undergoing any specific therapy.<br>A follow-up echocardiogram conducted on July 2, 2020, indicated the following findings:<br><br>Heart rate frequency ranges from 110 to 120 beats per minute.<br>The electric axis of the heart is positioned normally. |

| | | | |
|---|---|---|---|
| | In the left eye (OS), there is partial cloudiness and milder edema.<br>The lens in the right eye (OD) is transparent.<br>Due to a fleur in the left eye (OS), further assessment is not possible.<br>Upon fundus examination of the right eye (OD):<br>The optic nerve disc appears pale with sharp contours, reduced in size, and excavated by 0.4 due to glaucoma.<br>The course and caliber of the vessels appear normal.<br>The macula zone reflex is blurred, while the visible periphery appears normal.<br>The vascular bundle is misplaced.<br>Similarly, due to a fleur in the left eye (OS), further examination of the fundus is not possible. | | There is evidence of incomplete blockage of the right foot of the Gis bundle.<br>Signs of enlargement in the left ventricle are noted. |
| 2 y.o. and 6 month | In terms of visual acuity:<br>The right eye (OD) exhibits good spatial vision up to 60 cm.<br>The left eye (OS) has spatial vision limited to 20 cm.<br>Both eyes together (OU) show heterotropia, indicating misalignment of the eyes.<br>Extraocular movements are normal in all directions.<br>The corneas in both eyes (OU) show no negative changes, with a diameter of 10.5 mm.<br>Upon fundus examination of the right eye (OD):<br>The optic nerve disc appears pale with sharp contours, reduced in size, and excavated by 0.4 due to glaucoma.<br>The course and caliber of the vessels appear normal. | The electroencephalogram (EEG) conducted during both waking and daytime sleep indicates an immaturity in cortical rhythms. Sleep stages are characterized by low indices and reduced physiological sleep patterns. No signs of epileptic activity are observed.<br>The patient displays positive responses to parental care, reaching out for their hands. She is also able to follow certain requests and engage in activities such as passing a ball, dancing, and mimicking sounds and simple words in a syllabic structure. Currently, she is capable of rolling and throwing a ball, and is learning to open a chest using a button. While she can momentarily stand with support while holding a toy, she soon returns to crawling actively.<br>During the examination, diffuse muscle hypotension and reduced tendon reflexes are evident. | The patient's current condition is assessed as moderately severe for the primary condition, but it is stable. The patient is active and responds calmly to the examination.<br><br>Physical observations include:<br>Skin with a pronounced marble-like pattern.<br>Adequate and evenly distributed subcutaneous fat.<br>Joints appear normal externally, with full and painless range of motion.<br>Muscle tone is reduced.<br>Upon percussion over the lungs, clear pulmonary sounds are detected.<br>Auscultation reveals puerile (child-like) |

| | | | |
|---|---|---|---|
| | The macula zone reflex is blurred, while the visible periphery appears normal.<br>The vascular bundle is misplaced.<br>Similarly, due to a fleur in the left eye (OS), further examination of the fundus is not possible. | There is a noted unilateral neurosensory hearing loss. | respiration, with a respiration rate of 26 breaths per minute.<br><br>Visually, there are no apparent changes in the heart area. Heart sounds are rhythmic and audible, without any pathological noises. The heart rate is 112 beats per minute, and blood pressure is measured at 80/40 mmHg.<br>The abdomen is soft and allows for deep palpation without causing any pain. The liver is palpable at the edge of the rib arch, indicating normal positioning. The spleen exhibits normal characteristics.<br>Both bowel and bladder functions are reported as normal. |
| 3 y.o. | The patient's visual acuity is assessed as follows:<br>Right eye (OD): Spatial vision is effective up to 100 cm.<br>Left eye (OS): Spatial vision is limited to 30 cm.<br>Both eyes (OU) exhibit small-scale horizontal nystagmus and heterotropia, indicating involuntary eye movements and misalignment.<br>Extraocular movements in all directions remain intact.<br>Tonometry readings are as follows:<br>Right eye (OD): 20 mmHg<br>Left eye (OS): 24 mmHg<br>Regarding the corneas:<br>In the right eye (OD), there is buphthalmos, indicating enlarged corneal size, with a diameter of 12.5 mm.<br>In the left eye (OS), the cornea shows abnormal epithelium, subepithelial fibrosis, and opacity. | The patient does not exhibit any general cerebral or meningeal symptoms. Eye movement in all directions (upwards, downwards, sideways) is unrestricted. The mouth remains open, partly due to macroglossia (enlarged tongue), and there is hypersalivation. However, there is no chewing observed.<br>The patient demonstrates diffuse muscle hypotonia, along with reduced tendon reflexes.<br>In terms of motor skills:<br>She is actively crawling and has begun attempting to stand up without support, although she quickly returns to a seated position.<br>With the support of both hands, she can walk short distances.<br>While the patient does not make indicative gestures or request the use of a potty, she has not yet demonstrated the ability to identify body parts or | |

| | | | |
|---|---|---|---|
| | Upon examination of the right eye's (OD) fundus:<br>The fundus displays an albino coloration.<br>The optic nerve disc appears pale with sharp contours, reduced in size, and excavated by 0.4 due to glaucoma.<br>The course and caliber of the vessels are normal.<br>The macula zone reflex is blurred, while the visible periphery appears normal.<br>The vascular bundle is misplaced.<br>Similarly, examination of the fundus in the left eye (OS) is not possible due to a fleur. | objects in pictures. Additionally, she requires assistance while using a spoon.<br>Her vocabulary includes her first situational word, "no". She is beginning to call her parents and can use touch to attract attention. She also reaches out to her parents. Engaging in more complex play activities, she shows an interest in the sounds of toys.<br>Additionally, she enjoys dancing to music and finds enjoyment in observing herself in the mirror during playtime. | |

At the age of 2 years and 6 months, the girl displays the following orthopedic characteristics:

- She has a proportionate build and is capable of standing with support, as well as making stepping movements.
- However, she is not yet able to walk independently.
- Her head is positioned in the midline and can rotate both to the right and left.
- Forward and backward tilts are within the normal range.
- Facial symmetry is observed, and sternal, clavicular, and mastoid muscles on both sides are symmetrical, soft, and elastic.
- Both upper extremities are of equal length (D = S), with full and painless joint movements and hypermobility.
- The spine exhibits a kyphotic posture with weakened back muscles.
- The thorax features a wide rib appertaining.
- Lower extremity joints demonstrate full movement, with no difficulty in hip extension. Functional tests on hip joints yield negative results.
- Genu recurvation is present, and the gluteal and popliteal folds are symmetrical.
- Both lower extremities are of equal length (D = S), and the axis of the lower extremities is correct.

- The right foot displays a flat-valgus deformity with support on the inner edge of the foot, while the left foot is varus deformed with a weakened tibia peroneal group on the left.
